# “I take a deep breath first”: Adolescent self‐regulation and co‐regulation in contexts of urban poverty in Bogotá, Colombia

**DOI:** 10.1111/jora.70067

**Published:** 2025-08-21

**Authors:** María Cecilia Dedios Sanguineti, Valentina Yepes Fiallo, Shari Baddan Ortiz Sochandamandou, Nicolás Romero Bejarano, Crick Lund, Mark J. D. Jordans, Sara Evans‐Lacko

**Affiliations:** ^1^ School of Government Universidad de los Andes Bogotá Colombia; ^2^ Innovations for Poverty Action Bogotá Colombia; ^3^ Centre for Global Mental Health, Health Service and Population Research Department King's College London London UK; ^4^ Alan J Flisher Centre for Public Mental Health, Department of Psychiatry and Mental Health University of Cape Town Cape Town South Africa; ^5^ Care Policy and Evaluation Centre London School of Economics and Political Science London UK

**Keywords:** adolescence, co‐regulation, peers, poverty, qualitative, self‐regulation

## Abstract

Previous research shows that poverty can hinder the development of self‐regulation skills, yet little is known about how individuals living in poverty experience self‐regulation. This qualitative study aims to understand young people's experiences as they deploy self‐regulation strategies and navigate interpersonal and contextual barriers associated with chronic scarcity. Adolescents (*n* = 29) aged 10–19 years living in low‐income urban areas in Bogotá, Colombia, participated in semi‐structured interviews and provided journal entries. Data were analyzed using framework analysis. Findings reveal that adolescents self‐regulate using avoidance, reflection, physiologically mediated strategies, and self‐care practices. They regulate themselves to solve interpersonal conflict and achieve personal and family‐oriented goals. Co‐regulation practices primarily involving peers were identified. Despite having a rich repertoire of self‐regulation strategies, participants described that dysregulated responses by significant others and the lack of material resources interfere with their effective use of self‐regulation strategies. Our findings can inform interventions operating in contexts of adversity that seek to improve self‐regulation during adolescence, a critical age for preventing mental health conditions and adverse developmental outcomes.

## INTRODUCTION

Self‐regulation encompasses setting and pursuing goals while influencing one's emotional, cognitive, and behavioral states (Inzlicht et al., 2021). It includes the ability to use skills effectively and resist inappropriate responses (Zimmerman & Schunk, 2011). Improved self‐regulation has been linked to positive developmental and mental health outcomes, including higher academic achievement and reduced aggression, criminality, peer victimization, depression, anxiety, illicit drug use, and unemployment (Robson et al., 2020). Low‐income contexts challenge young people's self‐regulation‐related capabilities through exposure to chronic stress associated with poverty, marginalization, and adverse interpersonal experiences with those in their immediate social context exposed to the same challenges (McEwen & Morrison, 2013). Yet, the current evidence has overlooked the lived experience of adolescents living in these settings, particularly how youngsters adapt self‐regulation strategies in response to the environment and social interactions, and the goals young people seek to achieve through self‐regulation strategies.

Self‐regulation has two distinct levels: one involving the regulation of emotions, cognitions, and behaviors (Gross, 2015) and a second level involving the achievement of more distal goals that require a level of commitment in tandem with the regulation of cognitive, emotional, and behavioral states (Hamoudi et al., 2015; Pandey et al., 2018). Adolescence is a key developmental phase to study self‐regulation, as brain changes during this phase can affect self‐regulation capabilities. During adolescence, the prefrontal cortex‐implicated in self‐regulation functions including executive control, response evaluation, and modulation‐decreases its volume, whereas activity in the amygdala and ventral striatum, involved in more immediate emotional responses, increases (Palacios‐Barrios & Hanson, 2019). Congruently, the evidence shows that adolescents experience difficulties controlling impulses, delaying gratification, and managing social situations with self‐regulation skills (Murray et al., 2015).

The study of self‐regulation has been primarily focused on psychological skills, leaving aside a more thorough understanding of its interpersonal origins and social function (Conover & Daiute, 2017). Yet, studies on co‐regulation show that social interaction plays a key role in the development of regulatory processes, including emotional regulation (Dixon‐Gordon et al., 2015; Hofmann, 2014; Zaki & Craig Williams, 2013). Further, adolescence is a critical period to study self‐ and co‐regulation due to the influence of peers and significant others in shaping behavior and the brain changes occurring at this stage. Bandura's social cognitive theory (1986) is particularly useful to study the development of self‐regulation as it emphasizes learning through observation and modeling of significant others like family members, teachers, and peers, and the two‐way interactions between personal factors, behavior, and the environment. While self‐regulation and co‐regulation have been studied among children in learning situations in the school context (Hoyle & Dent, 2018), less is known about co‐regulation practices among adolescents, even though peer influence is at its peak during this developmental stage (Andrews et al., 2021).

### Self‐regulation in contexts of adversity

Developmental and neurobiological studies show that poverty has detrimental effects on self‐regulation and mental health through diverse pathways, including bottom‐up and top‐down processes (Palacios‐Barrios & Hanson, 2019). Substantial evidence suggests that prolonged scarcity can impair the development and use of self‐regulation skills primarily due to chronic stress (Mani et al., 2013; McEwen & Morrison, 2013). Contexts characterized by recurrent immediate demands and high rates of unpredictability are known to divert attention away from long‐term decision‐making toward short‐term responses (Hamoudi et al., 2015; Martínez & Maner, 2023; Mullainathan & Shafir, 2013). Self‐regulation and problem‐solving skills are impacted because these require considering the future implications of decisions. In turn, difficulties with self‐regulation hamper organization and planning, which interfere with goal achievement (McEwen & McEwen, 2017).

### Gaps in the literature on self‐regulation

Self‐regulation plays a critical role in individual and social functioning, yet key aspects have been overlooked because of how it has been operationalized in most empirical studies. Self‐regulation has been studied primarily as a cognitive task due to its close theoretical and empirical relationship with executive functions. Executive functions, together with working memory, inhibitory control, cognitive flexibility, and attentional control, allow individuals to regulate themselves, but self‐regulation is a broader phenomenon, including orientation toward achieving goals (Hamoudi et al., 2015; Pandey et al., 2018). The constructs of self‐regulation and coping have often been used interchangeably, which has obscured the understanding of the former. While self‐regulation encompasses coping strategies such as reappraisal, distraction, and support‐seeking aimed at dealing with internal and external demands (Fombouchet et al., 2024), it extends beyond the management of challenging situations, as it includes regulation aimed at achieving goals and the maintenance of well‐being. Also, the use of standardized measures, observational methods, and lab‐based tasks has rendered the study of self‐regulation as an all‐or‐nothing construct in which individuals have or lack the skills necessary to regulate themselves (Conover & Daiute, 2017). These conceptual and methodological approaches have given insufficient attention to the study of self‐regulation as a dynamic process that is closely connected to goal‐oriented behavior.

Though the relationship between poverty and self‐regulation has been well documented, a more thorough understanding of how chronic scarcity impacts young people's key relationships and immediate social environments is still lacking. Poverty has detrimental effects on interpersonal networks through poor social support, lack of safe living conditions, and violence, all contributing to increases in emotional distress and conflicts (Marshall Lee et al., 2022). At the family level, socioeconomic stressors may interfere with parenting practices and lead to conflict and aggression in the caregiver‐child relationship. This impacts the development of emotional processing networks, is correlated to externalizing behaviors in youth, and can further perpetuate aggressive responses (Vannucci et al., 2023; Wadsworth et al., 2013). In sum, adolescents living in contexts of poverty face a dual challenge; they have to navigate both their own recurrent contextual demands and the additional burden of others' dysregulation. The role of this double burden of self‐regulation requires further research, as it is not yet known how adolescents experience it, how they adapt their self‐regulation skills to this social context, and what barriers they encounter in the process. Lastly, more research is needed on how the sociocultural context, including cultural norms, community and familial relationships, and social structures, either facilitates or impedes self‐regulation. Understanding these dynamics is essential for developing effective self‐regulation interventions to support adolescents living in low‐income settings.

### Study aims

This study employs a qualitative approach to understand how adolescents living in low‐income settings in Colombia deploy self‐regulation skills across different contexts and social interactions. We define self‐regulation at two levels: the regulation of the individual's psychological states and the goals for which they regulate themselves. The research aims to describe young people's lived experiences of self‐regulation, including the strategies adolescents use for self‐regulation, the rationale for deploying specific strategies, and their accounts of how the sociocultural and environmental context either facilitates or impedes their ability to self‐regulate.

## METHODS

### The ALIVE project

This study was part of the research project “Improving Adolescent Mental Health by Reducing the Impact of Poverty” (ALIVE), aimed at understanding how poverty affects the most common adolescent mental health conditions directly and indirectly through its effects on self‐regulation (Lund et al., 2023). ALIVE seeks to develop and evaluate an intervention that aims to prevent depression and anxiety by strengthening self‐regulation and reducing poverty among adolescents living in urban poverty in Colombia, Nepal, and South Africa. The data reported in the current study builds upon the formative qualitative research in Colombia.

### Qualitative approach and design

This study is grounded in the phenomenological paradigm as it sought to understand adolescents' experiences of self‐ and co‐regulation (McWilliam, 2010). We employed a cross‐sectional design using semi‐structured interviews and diary entries to understand adolescents' lived experience of self‐regulation in low‐income settings in Colombia. The interviews and diaries allowed us to collect data on how adolescents experience self‐regulation across different situations, including conflicts at home, school, or with peers, and how they use their skills to achieve goals and maintain a state of well‐being. The qualitative approach allowed us to focus on the lived experience and practical knowledge participants have regarding self‐regulation, conflict resolution, and goal pursuit in low‐income contexts.

### Setting

The study was conducted in the Bogotá districts of Usme, Ciudad Bolívar, and Santa Fe. These were selected due to their high rates of multidimensional poverty. Multidimensional poverty goes beyond monetary poverty by assessing three dimensions impacting well‐being: monetary income, educational attainment and enrollment, and basic infrastructure services (World Bank, 2025). According to Colombia's National Administrative Statistics Department (DANE), Ciudad Bolívar, Usme, and Santa Fe are the districts with the highest rates of housing deficits in Bogotá and low access to public services (2021). These districts have internet access rates below the city's average of 80%: Ciudad Bolívar (64.8%), Usme (70.3%), and Santa Fe (73%). In addition, 38.96% of households in Ciudad Bolívar, 33.47% in Usme, and 29.18% in Santa Fe report that their incomes do not cover their minimum required expenses, which is consistent with subjective poverty rates, indicating that 45.7% of the inhabitants in Ciudad Bolívar, 44.6% in Usme, and 33.3% in Santa Fe described themselves as being poor. Regarding violence rates, 47% of the city's total crimes in 2023 clustered in four districts, two of which were Ciudad Bolívar and Santa Fe (Secretaría Distrital de Seguridad, Convivencia y Justicia, 2023). Income, household deficits, and violence rates are among the structural barriers that pose challenges to the inhabitants of these districts.

Ethnographic studies carried out in the neighborhoods of Ciudad Bolívar, Usme, and Santa Fe provide relevant information on community dynamics and interactions between residents. Ciudad Bolívar and Usme are among the districts receiving the highest numbers of internally displaced individuals from the armed conflict and former armed actors undergoing reintegration processes, resulting in accelerated demographic growth of formal and informal settlements (Ortega, 2021). A significant part of the residents' lives occurs outside their homes, yet the sense of security is low due to the presence of armed groups, paramilitaries, armed gangs, and drug dealers, who control areas delineated by invisible borders (Arrias, 2019; Medina, 2020; Pazos, 2012).

The sociocultural context is relevant to the study of self‐ and co‐regulation. Studies on collectivist and individualist cultures describe Latin American countries as collectivistic societies that emphasize collectivistic values while valuing a more independent self‐construal, encouraging autonomy, personal goals, and self‐expression, in contrast with the interdependent self‐construal fostered by collectivistic cultures in the East (Krys et al., 2022). There is emphasis placed on family interests, a hallmark of the *familism* culture prevalent in Latin America (Barbosa et al., 2023). Colombia's case is key for understanding the self in the cultural context, as it occupies a midpoint position between collectivistic and individualistic societies, according to the Global Collectivism Index (Pelham et al., 2022). Colombia exemplifies a complex interplay of collectivism and individualism, shaped by both cultural values centered around the family and autonomy of the self.

### Sample

We interviewed 29 participants aged 10 to 19 years. Participants were divided into two age groups: 11–14 years (age group 1) and 15–19 years (age group 2). As shown in Table 1, the sample was balanced in terms of gender, age, and district. This sample size aligned with other qualitative studies on self‐regulation (Collins & Durand‐Bush, 2010; Conover & Daiute, 2017).

**TABLE 1:** Participants' age and gender distribution in each site.

Demographics	Districts					
	Usme		Ciudad Bolívar		Santa Fe	
Age group	10–14	15–19	10–14	15–19	10–14	15–19
Male	3	3	2	3	1	2
Female	3	3	4	2	2	1

Participants' self‐reported life conditions indicate that many lived in poverty without access to public services, the internet, or a phone. Some of their caregivers did not have formal or stable jobs. Others were internally displaced by armed groups, with families having to start all over in the capital city. Some adolescents could not attend school because they could not afford to buy a uniform or school supplies. Urban gangs and drug availability were frequently mentioned as issues affecting their daily lives.

### Procedure

The research protocol was approved by the ethics review board at King's College London (Reference Number: HR/DP‐21/22‐27074) and Innovations for Poverty Action (Reference Number: 15584). The 3rd and 4th authors were trained to conduct the interviews, which included guidance on implementing the interview guide and activating the psychological first aid protocol. Both authors are native Spanish‐speaking researchers who are proficient in English. An on‐call psychologist was available during the data collection process to provide psychological first‐aid support when necessary and make referrals for participants who required or requested further psychological support.

Data collection was conducted in October and November 2022. Participants were identified based on the previous work of consortium partner, War Child Colombia, in these areas. The team at War Child, an international humanitarian organization, identified adolescents within the intended age range living in the three areas. The team was known to young people and adult community members from the previous programming implemented in these districts. Starting with former programming beneficiaries, a snowball sampling strategy was followed in each location. Informed consent was obtained from parents of underage participants, alongside the participants' informed consent. Participants 18 years and over provided informed consent before the start of any data collection. Each participant was assigned a unique identifier.

War Child scheduled the interviews through phone calls or home visits, as appropriate. In‐person interviews in Spanish were conducted at the participants' homes. In all cases, the research team guaranteed the participants' privacy by seeking a separate space to conduct the interview. Interviews were audio recorded and transcribed verbatim. Transcripts were anonymized, encrypted, and stored on safe servers. Data were analyzed by Spanish‐speaking researchers who were proficient in English. The coding frame that guided the analysis was developed in English.

### Instruments

A semi‐structured interview guide in Spanish was developed for this study (see Appendix A). The instrument sought to facilitate discussions about young people's knowledge and deployment of self‐regulation strategies in their daily lives. Interviews were structured around prompts referring to negative emotions elicited in the participant by relevant people (caregiver, teacher, friend) across three different social contexts (school, home, and peer group). An additional section explored the strategies participants used to reach goals. Participants were asked to develop stories based on these prompts, drawing from personal experience or that of peers. Prompts were unstructured to allow participants to elaborate on their experiences and provide details they considered relevant. To ensure participants were referring to self‐regulation experiences when narrating the stories, the instrument was designed to ask them specific questions about how they managed the emotions and solved the problem they were describing. Participants were further asked what they would advise a friend to do to manage their emotions when facing a similar situation. Questions were designed to explore the participants' repertoire of self‐regulation strategies, even if they described difficulties in implementing them.

Additionally, each participant received a journal at the end of the interview. Participants were asked to write journal entries at least once a week for a maximum of 4 weeks. Journal prompts referred to the past week and were about any adverse situation that may have happened, how they felt about these situations, and how they responded to them (see Appendix B). Telephone or in‐person follow‐ups were conducted at the beginning, middle, and end of the journal data collection period to remind participants about the task. Most (89%) participants completed the journal entries.

### Data analysis strategy

Diary data were considered cross‐sectional since the entries only referred to independent situations that had happened over the past week. Interviews and diary data were analyzed using the framework analysis methodology, which systematically identifies themes prevalent among the data using deductively predefined codes (Gale et al., 2013). The research team implemented the following framework analysis steps. First, to become familiar with the data, summary notes highlighting key information from the interviews and the journals were compiled. Subsequently, the team initiated the coding process using a deductive approach to identify self‐regulation practices, guided by the categories “behavioral self‐regulation” “cognitive self‐regulation” and “emotional/physiological self‐regulation” The coding frame was reviewed and refined through an iterative process by revisiting the summary notes, the interviews' transcriptions, and journal entries using an inductive approach. This yielded additional categories capturing the goals of self‐regulation, co‐regulation strategies, and codes to capture the role of violence on self‐regulation. The research team discussed modifications during regular meetings to harmonize the structure of the coding frame, being careful not to impose predefined concepts on the data and remaining open to newly identified codes. The final coding frame can be found in Appendix C (Table C1). The coding frame was applied to the entire dataset of transcripts in Spanish. The coding frame was then organized around the “*how*” and “*what for*” of self‐regulation categories, and the codes remained the specific strategies contributing to each category. Inter‐rater reliability, as measured by Cohen's Kappa coefficient, indicated a high level of agreement (86%) between two raters who coded data from 24% of the sample (*N* = 7), including interviews and diary entries (McHugh, 2012).

### Positionality

We are a group of researchers from different health and social science disciplines who share a common interest in studying the social determinants of mental health. We all share the core assumptions that mental health should not be studied in isolation from the broader context and that the study of lived experience through qualitative methods contributes to improving mental health theories and concepts. Local knowledge and experiences contribute to building evidence to enhance the effectiveness of interventions by challenging and enriching preconceived mechanisms of change.

Our team comprises seven researchers; three are based in academic and research institutions in Bogotá, Colombia, and four are employed by academic institutions in the Global North with extensive experience conducting research in LMIC countries. This team structure allowed us to combine the insider and outsider perspectives in interpreting the data (Berger, 2015), particularly on the aspects related to the cultural factors, by considering how interpersonal relationships influence the mechanisms yielding psychological well‐being and ill‐being. At the same time, we are not insiders to the experience of social vulnerability and growing up in low‐income contexts. Acknowledging the many potential blind spots and the importance of recognizing lived experience as a source of valid knowledge (Dedios Sanguineti et al., 2025), the ALIVE research project assembled a Youth Advisory Group (YAG). The YAG was consulted on the development of the interview guide and provided feedback on the study findings. YAG members confirmed that they use the self‐regulation strategies reported by the study participants, the importance of co‐regulation practices, and the interference of contextual barriers in deploying these strategies.

## RESULTS

Our analysis yielded detailed descriptions of the “*how*” and the “*what for*” of self‐regulation as experienced by study participants, and indicates the salience of the context in young people's daily lives. In describing self‐regulation, participants often mentioned interpersonal conflict with peers, teachers, parents, or siblings, financial difficulties, poor academic performance, and balancing work and study‐related responsibilities despite their young age.

### Self‐regulation strategies: how adolescents self‐regulate

As shown in Table 2, the data demonstrated four main categories of strategies for self‐regulation, namely (1) avoidance, (2) reflection, (3) physiological regulation, and (4) self‐care, alongside specific strategies young people use to regulate themselves. The last column in Table 2 depicts whether participants' descriptions of the specific strategy evidenced co‐regulation practices.

**TABLE 2:** Self‐regulation's how.

Self‐regulation strategies	Specific strategies	Co‐regulation
Avoidance	Engage in other activities	YES
	Take distance from interpersonal conflict	NO
Reflection	Perspective taking	YES
	Reappraisal	YES
	Spirituality	NO
	Self‐reflection	NO
	Draw from past experience	YES
Physiological regulation	Cry	NO
	Breathe	NO
Self‐care	Get engaged in rewarding activities	YES
	Self‐confident thoughts	NO

#### Avoidance

Participants frequently reported active avoidance of stressful situations. The two main avoidance strategies *were engaging in other activities* and *distancing themselves from interpersonal conflict*. The former involved engaging in activities to avoid thinking about the problem or dealing with the emotions caused by it. Examples included playing football, dancing, spending time with friends, walking, or sleeping. Some participants suggested that adolescents may use drugs to deal with stressful situations: “He [my friend] went to the street to be with his friends and use drugs to get rid of the anger” (male, age group 2). Adolescents mentioned risks associated with drug use, such as being thrown out of their homes. However, they perceived it as an effective short‐term strategy. Participants emphasized how these activities require networks and engaging peers in shared activities. This revealed a co‐regulation strategy deployed by youngsters who reached out to others to manage their emotions, thoughts, and behaviors.

A second avoidance strategy focused on dealing with interpersonal conflict and involved distancing themselves emotionally, cognitively, or physically from the situation. This strategy was deployed particularly when youngsters described feeling “hot‐headed” or experiencing emotions like anger or frustration. Taking distance allowed participants to regulate their emotions and avoid responding with verbal or physical aggression; hence preventing conflict escalation.

When one is like that, with a hot head, […] it is good to go to one side and take a moment, take a breath, so as not to mess it up later, because there are acts that go deep. (male, age group 2)

This strategy did not involve engaging in a specific activity but physically distancing themselves from where the conflict was taking place, as well as suppressing thoughts, emotions, and actions for a moment. Adolescents reported “calming down and waiting for time to pass before talking to the teacher” (male, age group 1). They also found it helpful to stay quiet, remain patient, count to 10, and let time pass to cope with their emotions.

Participants employed avoidance strategies in different ways to manage interpersonal conflicts. Some used avoidance strategies as an initial step to manage interpersonal conflict, allowing themselves to calm down before confronting the conflict directly. Others opted for avoidance as a more permanent solution to interpersonal conflict, particularly in situations deemed irresolvable. The latter was more common when past experience indicated they would fail at solving a specific problem, such as conflicts at home, where they learned that their parents were not open to dialogue or were abusive. For example, a female participant from age group 2 shared she decided to leave her home for a while and live with a friend with whom she used drugs to avoid the constant fights with her mother, who would tell her comments like “I should have aborted you, it would have saved me the headaches with you.”

#### Reflection

Participants described five strategies to think through challenging situations before deciding what to do, and to identify if a response is required from them at all. Adolescents try to understand the reasons or intentions behind others' actions to navigate interpersonal conflict or disagreement. For example, they used *perspective‐taking* when teachers scolded them for being distracted in class. They might initially feel upset, but then they calmed down by thinking that the scold could mean that “the teacher cared about the students' future and wanted them to learn” (female, age group 1). Alternative explanations could be proposed by themselves or by trusted others, revealing again the importance of co‐regulation. Participants reported using this strategy at home, as they perceived that their parents might reprimand them because they are tired rather than because they are upset with them.

Participants described *reappraising situations* by identifying the positive side in challenging situations, accepting things that cannot be changed, and moving on. Examples included failing an exam or breaking up with a romantic partner. A male participant from age group 1 comforted a friend after a breakup by telling him his former girlfriend “had been the girlfriend of half of the students in the school,” and hence he was better off now that the relationship had ended.

A third strategy was *spirituality*, consisting of interpreting reality from a perspective guided by religious beliefs or the existence of a greater order. This strategy reassured participants in their day‐to‐day challenges and decisions. Participants from age group 1 and 2, respectively, used expressions like “with God's help, things will turn out well” or “what is meant to be, will turn out well” when talking about the future or thinking about being accepted in a university. Young people's descriptions of spirituality were not a form of reappraisal because they did not always involve reframing a situation; these could be the first and only interpretation of events.

The fourth strategy, *self‐reflection*, means youngsters reflect on their own mistakes, strengths, and weaknesses, as well as their desires or hopes. Some of them used diaries as aids for self‐reflection. Self‐reflection allowed them to learn from past experiences, learn from their own mistakes, gain confidence in themselves, and deal with interpersonal conflicts; for instance, by apologizing or forgiving.

The last reflection strategy was to *draw from past experience*. Participants reported that reflecting on their current or past problems and mistakes helped them regulate their emotions and identify possible solutions based on previous experience. Drawing from past experiences was particularly important for participants to learn from mistakes related to acting impulsively or aggressively in similar situations. Oftentimes, close others helped them become aware of the learning potential of past mistakes.

These strategies often required participants to engage people in their support network. They sought peers to talk about the fights they had at home or talk to their parents about the problems they faced at school. Engaging others allowed them to vent, put words to their emotions, reframe situations, try to understand the other's point of view, and receive advice. A female participant from the age group 15 to 19 years old reported that she coped with conflicts with her boyfriend by “venting with someone else before talking to him.” These reports indicated a process of co‐regulation, where adolescents reached out to peers or adults for help in regulating emotions and guidance for dealing with complex interpersonal situations. The only strategies in which a process of co‐regulation was not identified were spirituality and self‐reflection.

#### Physiological regulation

Adolescents highlighted a clear relationship between their emotional states and physical responses, describing how *breathing* and *crying* helped them to calm down, specifically when feeling anger, frustration, sadness, and anxiety. Breathing was identified as a deliberate practice adolescents used to calm themselves down, while crying was more often described as an automatic response that served a cathartic function. Both breathing and crying helped participants regulate themselves. For example, when a female participant from age group 2 was asked how she dealt with high levels of anger due to infidelity, she said: “I cried. I feel that, yes, it's like one's way of venting”. Crying and breathing were described as individual regulation strategies. Participants reported advising their peers to breathe to avoid conflict escalation, but did not report breathing together with their peers, nor did they specify whether their peers adopted their advice.

#### Self‐care

Participants often reported engaging in self‐care practices to enhance well‐being and strengthen self‐confidence, which in turn contributed to self‐regulation. *Engagement in rewarding activities* was described as a self‐care practice, and listed activities included playing football, putting on makeup, dancing, and spending time with friends. These activities, enjoyable and valued, often overlapped with strategies for avoiding stress, yet in the context of self‐care, they served to actively reinforce personal well‐being. Adolescents engage in these practices individually or with others, highlighting the importance of the social network to their well‐being. Having *self‐confident thoughts* was a second form of self‐care. These thoughts helped adolescents boost their self‐esteem and encouraged them to pursue their goals. Self‐confident thoughts included “I can” statements of self‐love and pride in one's achievements. For example, a female participant from age group 1 mentioned facing many challenges throughout her life, including comments about her skin color and body shape. She found reassurance in thinking of herself as “a strong, capable, and intelligent woman who can show the world that she can accomplish things.” Participants repeated to themselves, “I can” to overcome obstacles and stay consistent in their goals. A female adolescent from age group 2 explained that she plans to become a nurse and thinks to herself, “I can do this, I can do this, I can do this” because otherwise, “I'll never be able to [keep motivated].” Self‐confident thoughts countered the punitive or self‐degrading self‐talk participants might engage in when they make a mistake.

### The ‘what for’ of self‐regulation

Table 3 depicts the self‐regulation goals reported by participants, along with the specific self‐regulated practices aimed at achieving the goals. These practices entailed a first level of self‐regulation as they required adolescents to inhibit impulsive responses, organize their thoughts, and stay calm, for which they built upon some of the strategies depicted in the *how* of self‐regulation (Table 2). Co‐regulation strategies were also present in this second level of self‐regulation because adolescents often reported involving others to help them attain their self‐regulation objectives of solving interpersonal conflicts and pursuing their goals.

**TABLE 3:** Self‐regulation's what for.

Self‐regulation goals	Self‐regulated practices	Co‐regulation strategies
Interpersonal conflict solving	Express emotions and opinions assertively	Third‐party mediation in conflicts and non‐material support in the form of advice
	Negotiate solutions through dialogue	
	Identify the best moment to talk	
	Ask for forgiveness/forgive	
	Set boundaries	
Goal pursuit	Set specific goals	Receive non‐material support
	Plan	
	Stay consistent	
	Have a purpose	

#### Interpersonal conflict solving

Participants described interpersonal conflict and aggression as part of daily life. Concerns included managing conflicts with parents and teachers and making amends after a fight with friends. Some youngsters framed conflict and aggression as the result of dysregulation, while others stressed that this is simply how interactions unfold around them. Aggression ranged from verbal insults to physical confrontations.

Participants talked about five self‐regulated practices for conflict‐solving: expressing emotions and opinions assertively, negotiating solutions through dialogue, identifying the best moment to talk about the conflict, asking for forgiveness/ forgiving, and setting boundaries. A female participant from age group 1 explained that she dealt with conflicts with her mom by “leaving her alone and letting her calm down” before “talking with her, to ask her what is going on and try to find a solution.” This participant's experience exemplified how adolescents use some of the self‐regulation strategies depicted in the previous section –in this case, taking distance from interpersonal conflict and perspective taking– to achieve the more long‐term objective of solving a conflict.

Co‐regulation became evident through the adolescents' reports of involving peers, parents, or teachers in conflict mediation or needing advice on how to best deal with interpersonal conflict. A male participant (age group 2) mentioned he helped solve a conflict between two friends by listening to each of them for several days, “joining both sides of the story, and then, advising them to talk, I mean, to give each other some time to think things through and try to recover the relationship if possible.” Participants frequently mentioned using their own knowledge about self‐regulation to assist others in regulating themselves. A female adolescent from age group 1 said she mediated conflicts between her boyfriend and his father in the same way she dealt with her own, that is, by talking about the problems calmly.

Not all participants employed all these strategies, and many were aware that not all strategies were equally effective in solving a problem in all contexts. Some adolescents referred more to the strategies related to finding the best moment to express themselves assertively, whereas others emphasized setting boundaries or negotiating solutions. Nevertheless, participants frequently reported difficulties using these strategies due to dysregulated aggressive responses in their immediate context. A female participant from age group 1 mentioned she gives her mother space to calm down before talking about the conflict, noting that otherwise, “my mother could hit me, and I could hit her back.” Another participant in the same age group remarked that aggression among her classmates reflected problems at home, stating, “What happens at home gets acted up at school.” Thus, dysregulated behaviors in the form of impulsivity and aggression in adolescents' social environment were recurrent barriers they identified in solving interpersonal conflicts.

#### Goal pursuit

Participants engaged in detailed discussions about their short‐term, medium‐ and long‐term goals, including both personal and group‐oriented goals for their families. Examples included passing an important exam, becoming a football player, earning a professional degree, providing economic support at home so their parents can rest, and building a house for the family. Goals were described as a source of motivation that helped them orient their decision‐making in their day‐to‐day lives. For instance, a female adolescent from age group 1 expressed that her greatest source of pride was having been able to help her mother build a house for herself and five other relatives, as they had migrated from another country and did not have a place to live. She highlighted that this was possible because of her mother's effort in her job as a recycler and because of the hard work, commitment, and motivation she and her relatives put into this task. A female adolescent from age group 2 mentioned having the goal of getting into Colombia's most prestigious public university. She emphasized the significant effort, preparation, and consistency this goal required due to the limited number of slots and other responsibilities she had to manage while preparing for the admission exam:

The preparatory course for the university exam started very early in the morning; I came back at 3 pm to work until 9 pm and then did some homework for the course. That is the way it was every day.

To achieve their goals, participants deploy four main strategies: setting specific goals, planning, staying consistent, and having a purpose. Planning the steps to achieve goals and staying consistent in the process allowed them to assess the effort required to keep themselves on track and overcome self‐identified obstacles such as ‘laziness.’ Having a purpose, described as value‐oriented principles like helping others or being good sons, daughters, or parents, helped youngsters to persist in pursuing a goal. “My motivation is my son […] and my goal is to pursue the career I have always wanted. Well, also to get ahead” (age group 2 female participant).

Regarding co‐regulation, peers, parents, and teachers played an important role in goal achievement, as adolescents stressed the importance of additional support to stay firm in their plans. Adolescents engaged networks to receive either material (i.e., economic) or non‐material support. Co‐regulation practices were evident in the provision of non‐material support, as this implied reaching out to relevant others to sustain self‐regulation. A male participant from age group 2, whose goal was to be a professional football player, said his family support has been a key motivating factor:

My dad got me into a football school since the beginning and has been supporting me since then, my mom is the one that always accompanies me to the games and the one that is always supporting me, as well as my sister […] my family has been keeping me motivated, and it would be great that one day I can be in a stadium knowing my family is there watching me.

Co‐regulation between peers worked both ways; adolescents sought out and supported their peers, acting upon an expectation of reciprocity in their relationships with significant others. A male participant (age group 1) told us he supports his football teammates whenever they get bullied after making a mistake by telling them that “we're not born knowing everything” to encourage them to keep practicing instead of losing motivation.

Lastly, participants mentioned self‐care strategies to manage self‐doubt and laziness. A male participant from age group 2 told himself, “I'm going to play well, I'm going to do this!” whenever he felt nervous in a football match. Repeating “I can” to themselves helped them increase their motivation and persist in achieving their goals. However, structural barriers related to poverty also affected the achievement of goals. Many participants reported not having the economic resources to pay for the dancing or football classes they wanted or the required uniforms. Others mentioned not having enough time to study due to responsibilities at home or in their after‐school jobs. Finally, some participants shared that they aspired to obtain a professional degree but were either not currently enrolled in school or had experienced periods of being out of school due to a lack of economic resources to cover transportation, uniforms, and other necessary expenses.

## DISCUSSION

Our study sought to understand the lived experience of self‐regulation among adolescents living in low‐income settings in Bogotá, Colombia. The findings offer rich descriptions of self‐regulation's ‘how’ and ‘what for’ and reflect self‐regulation's individual and social dimensions. Youth rely on many effective self‐regulation and co‐regulation strategies to solve interpersonal conflicts and attain individual and shared goals. This dual aspect of self‐regulation underscores its complexity and highlights the importance of social interactions and the context in shaping self‐regulatory behaviors, as predicted by Bandura's social cognitive theory (1986). In line with Conover and Daiute's findings (2017), participants demonstrated having a thorough knowledge of how to self‐regulate but were sometimes unable to apply this knowledge effectively. Poverty and violence not only constrained adolescents but also influenced their immediate social context, including key actors like caregivers and teachers. These barriers stemming from cumulative and acute exposure to adverse contexts impede adolescents' ability to manage conflicts and pursue goals. Adolescents reported failing to solve conflicts with their parents, teachers, and peers, not because they did not know how to handle the situation, but because significant others were often stressed and reactive. Similarly, participants reported failing to achieve goals despite deploying self‐regulation skills because of a lack of economic resources. The chronic stress experienced by individuals living in adverse contexts impacts the development of self‐regulation skills through the effects of deprivation and threat (McCoy, 2013; Palacios‐Barrios & Hanson, 2019). At the same time, immediate contextual barriers further impede the implementation of self‐regulation strategies. Our findings reveal that the barriers of low‐income settings interfere with self‐regulation through various mechanisms beyond the sole hypothesis of the underdevelopment of individual self‐regulation skills and show how self‐regulation is deeply intertwined with others and the social context.

In line with Bandura's social cognitive theory (1986), a main finding of this study is that adolescents commonly engage in co‐regulation practices with parents, teachers, and peers. This finding echoes a large body of research highlighting the importance of emotional, cognitive, and behavioral co‐regulation, which involves others through socially mediated processes like modeling and perspective‐taking (Hofmann, 2014; Nozaki & Mikolajczak, 2020; Zaki & Craig Williams, 2013). Research on co‐regulation has primarily focused on the caregiver‐child relationship (Murray et al., 2015; Spinrad et al., 2020) and the teacher‐student relationship (Opdenakker, 2022). Our study results support previous findings and expand on them by describing the experience of adolescents' co‐regulation practices with their peers across different self‐regulation strategies and objectives, showing that co‐regulation practices are used at the two levels of self‐regulation: to regulate their internal states and to pursue goals.

Peer influence during adolescence is often discussed in terms of its potential negative effect on adolescent behaviors (Dishion & Tipsord, 2011), decision‐making (Andrews et al., 2021; Chein et al., 2011), and overall self‐regulation skills via social rejection (King et al., 2018). Little is known about the enhancing potential of peer influence on self‐regulation described in our study. This positive aspect is especially significant in collectivistic cultures, prevalent in Latin American countries, where interpersonal relationships and community bonds are critical in shaping behaviors and developmental outcomes. Research indicates that collectivistic cultures differ in the use of self‐regulation strategies, with socially mediated strategies such as modeling and perspective‐taking being more widespread in these contexts (Liddell & Williams, 2019; Ramzan & Amjad, 2017; Trommsdorff, 2009). Colombia shares a combination of individualistic and collectivistic traits, with an emphasis placed on the family sphere (Barbosa et al., 2023; Krys et al., 2022). The family's relevant role is evident in the “*what for*” of self‐regulation, as many of the goals described by adolescents were family‐oriented goals. Moreover, adolescents described the support by family and friends as key for achieving goals and reported finding meaning in helping their close social networks. We hypothesize that the importance of the family and the peer sphere in self‐ and co‐regulation has a dual explanation: Colombia's cultural context and the peer influence operating at this developmental stage. Thus, interventions can be tailored to harness peer influence and family relations to facilitate self‐regulation.

Lastly, our findings suggest that self‐care practices used by adolescents to enhance well‐being and self‐confidence could promote self‐regulation and help adolescents stay on track to reach their goals. This is especially important in low‐income settings, where structural barriers related to scarcity, violence, and stress challenge adolescents daily. Research on youth resilience in disadvantaged settings indicates that close and supportive relationships with caregivers, teachers, and peers, alongside a strong sense of identity in which adolescents recognize themselves as competent, are important protective factors in contexts of adversity (Ungar, 2015). Our findings show how young people actively engage in rewarding activities alongside significant others and the value they find in self‐care practices as contributors to their well‐being. Interventions aiming to improve self‐regulation in contexts of adversity could approach well‐being not only as an outcome but as a mediating factor that facilitates self‐regulation, particularly under contextual adversity.

### Limitations

It is important to consider the limitations of our study. First, self‐regulation has a non‐linear evolution throughout adolescence, varying across early, middle, and late adolescence (Fombouchet et al., 2024; Murray et al., 2015). Because we lacked a large enough sample size, we could not track these variations across different age groups. A comparison across ages could have shed light on the developmental pathway of self‐regulation and co‐regulation strategies in adolescents living in low‐income settings. Participants were divided into two age‐groups, 10–14 and 15–19 years, but we did not collect their exact ages, which further interfered with a cross‐age comparison. Second, our instrument was overly focused on self‐regulation strategies, with less in‐depth exploration of self‐regulation goals and the contextual barriers to self‐regulation. Because goal setting is a key aspect of self‐regulation, future research should explore more openly the range of goals young people may have in low‐income settings and how they relate to their self‐regulation and co‐regulation strategies. Lastly, our study focused on the adolescents' perspectives. Yet, the identified relevance of co‐regulation practices requires that future studies include in their design the perspectives of significant others, such as parents, teachers, and peers, to further understand the interpersonal dynamics of co‐regulation.

### Implications and future directions

The study findings show that adolescents activate internal and external resources to navigate the challenges of contexts with high poverty levels. They have thorough knowledge about how to use different regulation strategies, when to use them, and how to get support from others for co‐regulation and goal achievement. Interventions to strengthen self‐regulation in low‐income settings should address the complex way adversity impacts self‐regulation. A dual focus on contextual barriers to deploying self‐regulation strategies and the impact of adversity on significant others supporting young people's self‐regulation should be considered in the interventions' mechanisms of change. As such, interventions should focus on strengthening self‐regulation skills while engaging significant others (e.g., parents, caregivers, peers, and teachers) involved in co‐regulation and on addressing more directly structural conditions such as poverty, lack of opportunities, violence, and drug use.

An important finding of this research is the recurring theme of violence, especially domestic violence, such as verbal aggression and degrading comments. This represents an additional burden for adolescents living in disadvantaged settings. To address this, self‐regulation interventions should focus on strengthening parental self‐regulation skills and parenting styles that can, in turn, prevent intergenerational cycles of violence. This would also contribute to better supporting the development of self‐regulation in adolescents by providing opportunities to engage in co‐regulation with their caregivers and learn self‐regulation skills through modeling.

Finally, future studies should explore peer‐based co‐regulation strategies in more depth. Co‐regulation practices can be taught and reinforced through small group interventions in schools, sports clubs, cultural centers, and other relevant spaces where adolescents interact with peers. Interventions and policies should focus on promoting co‐regulation practices among peers to enhance adolescent self‐regulation strategies.

## CONCLUSION

Our study provides an in‐depth account of adolescents' experiences of self‐regulation and co‐regulation in low‐income settings in Colombia. It offers insights into how poverty interferes with self‐regulation through various mechanisms, challenging explanations that attribute these difficulties solely to the underdevelopment of individual self‐regulation skills. Mechanisms include the negative impact of scarcity on young people's key relationships and immediate social environments, and economic barriers to attaining goals. Interventions targeting self‐regulation in similar populations could benefit from including in the theory of change a dual focus on building individual skills for self‐regulation strategies and addressing the impact of adversity on significant others (e.g., caregivers, peers, and teachers) who provide the scaffold for adolescents' developing self‐regulation skills.

## AUTHOR CONTRIBUTIONS

Conceptualization: Crick Lund, Mark J. D. Jordans, Sara Evans‐Lacko and María Cecilia Dedios Sanguineti. Methodology: María Cecilia Dedios Sanguineti, Nicolás Romero and Sara Evans‐Lacko. Data curation: Nicolás Romero. Investigation: Shari Baddan Ortiz and Nicolás Romero. Validation: Valentina Yepes Fiallo. Formal analysis: María Cecilia Dedios Sanguineti and Valentina Yepes Fiallo. Funding acquisition: Crick Lund and Mark J. D. Jordans. Writing – original draft: María Cecilia Dedios Sanguineti and Valentina Yepes Fiallo. Writing – review and editing: María Cecilia Dedios Sanguineti, Valentina Yepes Fiallo, Shari Baddan Ortiz, Nicolás Romero, Crick Lund, Mark J. D. Jordans and Sara Evans‐Lacko.

## FUNDING INFORMATION

This work was supported by the National Institute for Health Research (NIHR), using the UK's Official Development Assistance (ODA) Funding, and Wellcome (grant number: 221940/Z/20/Z) under the Department of Health and Social Care (DHSC)‐Wellcome Partnership for Global Health Research. The views expressed are those of the authors and not necessarily those of the Wellcome Trust, NIHR, or the DHSC.

## CONFLICT OF INTEREST STATEMENT

The authors have no conflicts of interest to declare.

## ETHICAL APPROVAL STATEMENT

The research protocol was approved by the ethics review boards at King's College London (Reference Number: HR/DP‐21/22‐27074) on April 22, 2022, and Innovations for Poverty Action (Reference Number # 15584) on May 24, 2022.

## PATIENT CONSENT STATEMENT

Participants 18 years old and over provided their informed consent, and underaged participants provided their informed ascent to participate alongside their caregivers' informed consent. Each participant was assigned a unique identifier to ensure anonymity.

## Data Availability

The data that support the findings of this study are available on request from the corresponding author. The data are not publicly available due to privacy or ethical restrictions.

## APPENDIX A Semi‐structured interview guide

For each of the three situations, interviewers were asked to follow the steps below. They were asked to start with the first story and complete all the steps before continuing with the next one.

**Situation 1: At school, I got angry with my teacher**

**Situation 2: At home, my mom/guardian/caregiver is making me feel very bad**

**Situation 3: With friends, my friend hurt my feelings**

***Step 1: Tell a story***

Read the first situation to the participant slowly and clearly. Once you have finished, ask if they want it reread. Ask them to create a story around this scenario, giving a name to the character in the story. You can use the following questions to get context, emotions, thought processes, and behavioral reactions from the participants.

Consider the following questions when directing the interview:

- Has this ever happened to you? To any of your friends or classmates? (Allow time to identify the story and the character involved.)
- To begin with, can you tell me what happened to that person that led to that reaction?
- What might they have said or done? Who was involved?
- What do you think he/she (referring to the character) was feeling when it happened?
- What do you think he/she (referring to the character) was thinking when it happened?

Delve into the explanation of the story, what emotions they felt, and what they were thinking when it happened.

- If the story is about a friend, what do you think caused these problems and situations at school for [name]?
- Inquire if the participant has difficulty responding, for example, if the problems were with their teachers, friends, or parents. What kind of problems? What do you think happened before [the problem] that caused it?

***Step 2: If the story is about someone else, make it about the participant themselves***

Let's imagine that you are the person in that situation instead of [name].

- If it were you, how do you think you would feel?
- What would you do if you were the person in that situation?
- How would you react?

**Step 3: Reflecting on a friend or sibling**.

Now I'm going to ask you to imagine that your friend or your sibling is the one in this situation, not you. If it were your friend or sibling,

- What would you tell them to do?
- Why would you tell them to do it?
- What would happen if they followed your advice?

***Step 4: Compare your answers***

- Now let's compare your answers when we imagined you were the one in this situation; when it was your friend or sibling.
- Do you notice any difference in how you would have reacted and how you would have advised your friend or sibling to react?

If necessary, explain: remember you said you would do [REPEAT ANSWER] if it were you; but you said you would advise your friend or sibling to do [REPEAT ANSWER]. What are the differences between these two strategies/responses to the situation? What do you think about these differences? Why do you think they are different?

For all the previous steps, if the person finds it difficult to construct the story, provide names, set the context. Keep in mind to develop questions that allow people to reflect on topics that are of interest to us and to expand on their response (where, how, who, then, etc.). Ask questions that facilitate the identification of self‐regulation mechanisms, which is our goal. Some examples include:

- Do you apply the advice you would give to your friends?
- How do you solve the problems you're talking to me about?
- How do you deal with your emotions?

**Situation 4: I achieved a goal I had been pursuing for a long time (for example, related to sports, school activities, an exam, music, helping someone achieve what they wanted, etc.)**

- What kinds of things helped them achieve the goal?
- Were there things that got in the way and made it difficult to achieve their goal? (Use this question to determine representations of poverty, considering indicators of multidimensional poverty: access to education, health, housing, and income)
- How did they stay motivated throughout the process?

If the person finds it difficult to construct the story, provide names, set the context. Keep in mind to develop questions that allow people to reflect on topics that are of interest to us and to expand on their response (where, how, who, then, etc.). Ask questions that facilitate the identification of self‐regulation mechanisms, which is our goal. Some examples include:

- Do you apply the advice you would give to your friends?
- How do you solve the problems you're talking to me about?
- How do you deal with your emotions?

**Situation 5 (optional): Poverty scenario**
I went to school with a torn uniform or without a uniform because I didn't have one…
I went to bed hungry the night before because there was no food in the house…
I don't have a phone, so I can't access social media…

Position the participant within this situation and consider the highlighted elements to create a story. Place the participant within this situation and consider the highlighted elements to create a situation and reflection around this case of poverty.

## APPENDIX B Journal entry guide

For the journal entries, we want to know about the challenges you face in your life, or something that bothered or angered you this week, whether at home, at school, or with your friends. For example, if you got into trouble at school or had a fight with your friends or family.

We will ask you to write your thoughts on some questions. These will be the same questions we asked you during the interview.

- What challenge did you face this week? Or what was something that bothered or angered you? Please describe what happened to you.
- How did you respond to/react to this situation? What did you do because of this challenge?
- How did you feel/what thoughts did you have because of this situation?
- If this happened to your brother or sister or a friend, how would you tell them to respond? What would you tell them to do?

Why are we making these journal entries? We want to gather real‐life examples of challenging experiences. We also want to learn from you about how to deal with these challenging experiences. Your participation will help make the program we are developing more realistic and useful for teenagers.

## APPENDIX C Coding frame

The columns “Self‐regulation categories” and “Specific practices” reflect the two layers of strategies participants reported using to regulate themselves. The four self‐regulation strategies in the “How of self‐regulation” section are the first four categories of practices and the corresponding specific practices. The next two categories of practices, “Assertive communication” and “Objectives,” with their corresponding specific strategies, coincide with the self‐regulation's *what for*, depicted in the second section of the results. The last self‐regulation categories, “Network activation” and “Aggression,” helped inform co‐regulation dynamics as well as the high prevalence of aggressive interactions in the participants' context. These two categories were useful in understanding the barriers to self‐regulation strategies and how participants activated interpersonal networks to get support, both through co‐regulation practices and seeking material support.

**TABLE C1:** Coding frame.

Self‐regulation categories	Specific practices	Comments
Avoidance	Substance use	Included in the results section
	Distancing	
	Engage in other activities	
Reflection	Perspective taking	Included in the results section
	Reappraisal	
	Spirituality	
	Self‐reflection	
	Draw from past experience	
	Think/talk about situations	
Physiological regulation	Cry	Included in the results section
	Breathe	
	Sleep	Included in “Engage in other activities”
	Time‐out	Included in “Distancing”
Self‐care	Get engaged in rewarding activities	Included in the results section
	Self‐confident thoughts	
Assertive communication	Express emotions and opinions assertively	Included in the results section
	Negotiate solutions through dialogue	
	Identify the best moment to talk	
	Ask for forgiveness/forgive	
	Set boundaries	
Objectives	Set specific goals	Included in the results section
	Plan	
	Stay consistent	
	Have a purpose	
Network activation	Seek support from institutions	Informed co‐regulation strategies across self‐regulation's *how* and *what for* strategies
	Seek peer support to solve conflicts with adults	
	Seek peer support to solve conflicts with peers	
	Seek adult support to solve conflicts with adults	
	Seek adult support to solve conflicts with peers	
	Give material support	
	Receive material support	
	Give non‐material support	
	Receive non‐material support	
Aggression	Physical self‐aggression	Informed findings regarding aggression and its interference with self‐regulation's *how* and *what for* strategies
	Verbal self‐aggression	
	Physical hetero‐aggression	
	Verbal hetero‐aggression	
	Retaliation	
